# Supplementary catalogue of the Anthomyiidae (Diptera) of China

**DOI:** 10.3897/zookeys.453.8282

**Published:** 2014-11-11

**Authors:** Mengmeng Wang, Verner Michelsen, Kai Li, Weibing Zhu

**Affiliations:** 1 School of Life Science, East China Normal University, Shanghai, 200062, China; 2 Institute of Plant Physiology and Ecology; Shanghai Institutes for Biological Sciences; Chinese Academy of Sciences; Shanghai, 200032, China; 3 Natural History Museum of Denmark (Zoological Museum), University of Copenhagen, Denmark

**Keywords:** Nomenclature, classification, Anthomyiidae, Scathophagidae, China, Taiwan

## Abstract

The present catalogue of Anthomyiidae attempts to list all species (173) described or recorded from mainland China (165) and Taiwan (8) that for various reasons are not treated in “Flies of China” from 1998. The catalogue further lists Chinese species that are presently standing in new generic combinations compared to those of “Flies of China”, species that have changed name because of synonymy or misidentification, and species upgraded from subspecies to species. Regional distribution by province is specified for all species. Literature sources to descriptions or records of anthomyiid species from China are only given for those 173 species not covered by “Flies of China”. Four new combinations are proposed: *Enneastigma fulva* (Malloch, 1934), *Enneastigma henanensis* (Ge & Fan, 1982), *Enneastigma lengshanensis* (Xue, 2001) and *Hylemya qinghaiensis* (Fan, Chen & Ma, 1989). *Eremomyia turbida* Huckett, 1951 is revived from synonymy with *Chortophila triticiperda* Stein, 1900 (current name *Eutrichota turbida*). One subspecies is upgraded to species: *Adia asiatica* Fan, 1988. The following eight new synonymies are proposed: *Delia pectinator fuscilateralis* Fan in Fan & Zheng, 1992 with *Delia pectinator* Suwa, 1984; *Eremomyia pilimana pilimarginata* Fan & Qian in Fan, Chen, Ma & Ge, 1982 with *Eremomyia turbida* Huckett, 1951 (current name *Eutrichota turbida*); *Lopesohylemya* Fan, Chen & Ma, 1989 with *Hylemya* Robineau-Desvoidy, 1830; *Deliomyia* Fan in Fan et al., 1988 with *Subhylemyia* Ringdahl, 1933; *Hydrophoria disticrassa* Xue & Bai, 2009 with *Hydrophoria pullata* Wu, Liu & Wei, 1995 (current name *Zaphne pullata*); *Heteroterma* Wei, 2006 with *Scathophaga* Meigen, 1803; *Heteroterma fanjingensis* Wei, 2006 with *Scathophaga curtipilata* Feng, 2002; *Scatomyza fansipanicola* Ozerov in Ozerov & Krivosheina, 2011 with *Scathophaga curtipilata* Feng, 2002. The genus *Heteroterma* Wei, 2006 and species *Heteroterma fanjingensis* Wei, 2006 are reassigned from Anthomyiidae to Scathophagidae.

## Introduction

China is a huge country of 9.6 million square kilometres in eastern Asia supporting a rich Palaearctic biota supplemented with a smaller Oriental biota in the southern areas. The Anthomyiidae are a large and diverse family of muscoid Diptera with c. 2000 described species worldwide. Anthomyiid flies are most diverse under temperate to subarctic conditions in the Northern Hemisphere. Accordingly, the Chinese anthomyiid fauna is exceedingly rich, including about one-third of the known world fauna, but still far from exhaustively investigated despite the voluminous literature on the subject.

The first comprehensive revision of the Anthomyiidae in China is that of Fan et al. (1988) who recognized 352 named species/subspecies in 43 genera. A second, more summary treatment of the family was given by Wei et al. (1998a, b) in the monumental “Flies of China” issued in two volumes (Xue and Chao 1998a, b). They recognized 515 species/subspecies in 43 genera from mainland China (514) and Taiwan (1). This figure has presently been adjusted to 511 species because of subsequent synonymy proposals. After combining this number with the extra species from the present catalogue, the number of anthomyiid species recorded from China has now reached 684 species in 36 genera. This number includes 9 species recorded from Taiwan but not from mainland China. The lower number of genera reflects recent attempts toward a phylogeny-based classification of the family in terms of supposedly monophyletic genera.

The notable increase in number of anthomyiid species presently recognized from mainland China (675) compared to “Flies of China” (511) reflects on one hand the high activity level of taxonomic and faunistic investigation of anthomyiid flies that has taken place in China since 1992/93 (the approximate deadline for “Flies of China”). However, our catalogue includes 25 species described as new and 17 species recorded from China or Taiwan before 1992 that for unknown reasons are omitted from “Flies of China”. These aspects emphasize the strong need of the present supplementary list.

## Materials and methods

The following catalogue is primarily a compilation of all anthomyiid species recorded from mainland China and Taiwan but for various reasons omitted from consideration in “Flies of China” (Wei et al. 1998a, b). These species are marked with an asterisk. The catalogue further includes species that (1) are presently recognized in a different generic combination, (2) have changed name because of synonymy or misidentification, or (3) have been upgraded from subspecies to species rank.

The arrangement of the species is alphabetical by genus and by species. The generic classification follows the most recent update of the family in “Fauna Europaea” (Michelsen 2011). Accordingly, all taxa ranked as subgenus or subspecies in “Flies of China” are either upgraded to genus or species rank or synonymized. Synonyms are only given to the extent that these are treated as valid genus or species group names in “Flies of China”. A reference to the original Chinese record is given for all anthomyiid species in the catalogue that have not received treatment in “Flies of China”. The known distribution in China by province given for all species included in the catalogue. An explanatory ‘Taxonomic note’ is added whenever the present nomenclature deviates from that of “Flies of China”.

## Catalogue

**Genus *Adia* Robineau-Desvoidy, 1830**

*Adia asiatica* Fan in Fan et al., 1988, STAT. REV.

*Adia grisella asiatica* Fan. Wei et al. 1998a: 670.

Distribution in China. Neimenggu, Qinghai, Sichuan, Yunnan.

Taxonomic note. The present taxon differs significantly from *Adia grisella* (Rondani) in the structure of the male terminalia. It also has a distribution overlapping with *Adia grisella* in Central Asia (DM Ackland in litt.).

**Genus *Alliopsis* Schnabl & Dziedzicki, 1911**

Syn.: *Paraprosalpia* Villeneuve, 1922.

Taxonomic note. Synonymy first proposed by Michelsen (1985: 39).

*Alliopsis billbergi* (Zetterstedt, 1838)

*Paraprosalpia billibergi* [misspelling of *billbergi*] *shanghaina* Fan in Fan, Chen, Cui & Wang, 1983.

*Paraprosalpia billergi* [misspelling of *billbergi*] *shanghaina* Fan. Wei et al. 1998a: 760.

Distribution in China. Heilongjiang, Shanghai.

Taxonomic note. Synonymy first proposed by Michelsen (2004).

*Alliopsis denticauda* (Zetterstedt, 1838)

*Paraprosalpia denticauda* (Zetterstedt). Wei et al. 1998a: 760.

Distribution in China. Heilongjiang.

*Alliopsis flavipes* (Fan & Cui in Fan, Chen, Cui & Wang, 1983)

*Paraprosalpia flavipes* Fan & Cui. Wei et al. 1998a: 760.

Distribution. China: Heilongjiang.

*Alliopsis maculifrons* (Zetterstedt, 1838)

*Paraprosalpia lutebasicosta* Fan in Fan, Chen, Cui & Wang, 1983.

*Alliopsis lutebasicosta* (Fan). Wei et al. 1998a: 756.

Distribution in China. Heilongjiang.

Taxonomic note. Synonymy first proposed by Michelsen (2004)

*Alliopsis magnilamella* (Fan in Fan, Chen, Cui & Wang, 1983)

*Paraprosalpia magnilamella* Fan. Wei et al. 1998a: 760.

Distribution. China: Qinghai.

*Alliopsis moerens* (Zetterstedt, 1838)

*Paraprosalpia moerens* (Zetterstedt). Wei et al. 1998a: 760.

Distribution in China. Heilongjiang, Neimenggu.

*Alliopsis pilitarsis* (Stein, 1900)

*Paraprosalpia pilitarsis* (Stein). Wei et al. 1998a: 760.

Distribution in China. Heilongjiang, Xinjiang.

*Alliopsis sepiella* (Zetterstedt, 1845)

*Paraprosalpia sepiella* (Zetterstedt). Wei et al. 1998a: 760.

Distribution in China. Xinjiang.

*Alliopsis tibialis* (Fan & Wang in Fan, Chen, Ma & Ge, 1982)

*Paraprosalpia tibialis* Fan.

*Parprosalpia* [misspelling of *Paraprosalpia*] *tibialis* Fan & Wang. Wei et al. 1998a: 760.

Distribution. China: Shanxi.

**Genus *Anthomyia* Meigen, 1803**

Syn.: *Craspedochoeta* Macquart, 1851.

Taxonomic note. Synonymy first proposed by Michelsen (1985: 39).

*Anthomyia alishana Ackland & Suwa in Ackland, 1987

Distribution in China. Taiwan (Ackland 1987: 46).

*Anthomyia cannabina* (Stein, 1916)

*Craspedochoeta cannabina* (Stein). Wei et al. 1998a: 651.

Distribution in China. Heilongjiang, Liaoning.

*Anthomyia confusanea* Michelsen in Michelsen & Báez, 1985

*Craspedochoeta liturata* (Robineau-Desvoidy, 1830) [misidentification]. Wei et al. 1998a: 651.

Distribution in China. Heilongjiang, Neimenggu, Shanxi, Xinjiang.

Taxonomic note. *Anthomyia liturata* (Robineau-Desvoidy, 1830) is known exclusively from Europe, whereas *Anthomyia confusanea* is a more widespread Palearctic species.

*Anthomyia hirsuticorpa (Feng & Fan in Feng, Fan & Zeng, 1999)

*Craspedochoeta hirsuticorpa* Feng & Fan.

Distribution. China: Sichuan (Feng et al. 1999: 321).

*Anthomyia lasiommata Fan & Chen, 1992

Distribution. China: Hainan (Fan and Chen 1992: 197).

*Anthomyia latifasciata Suwa, 1987

Distribution in China. Guizhou (Wei 2006b: 525).

*Anthomyia mimetica* (Malloch, 1918)

*Craspedochoeta angulata* (Tiensuu, 1938). Wei et al. 1998a: 651.

Distribution in China. Heilongjiang, Liaoning.

Taxonomic note. Synonymy first proposed by Michelsen (2004).

*Anthomyia oculifera* Bigot, 1885

*Anthomyia koreana* Suh & Kwon, 1985. Wei et al. 1998a: 648.

Distribution in China. Liaoning.

Taxonomic note. Synonymy first proposed by Griffiths (2001: 2256).

*Anthomyia psilommata Fan & Chen, 1992

Distribution. China: Hainan (Fan and Chen 1992: 198).

*Anthomyia pullulula* (Fan in Fan, Chen, Ma & Wu, 1984)

*Craspedochoeta pullulula* Fan. Wei et al. 1998a: 651.

Distribution in China. Shanxi, Qinghai, Xinjiang.

*Anthomyia sinensis Zhang & Sun, 1997

Distribution. China: Liaoning (Zhang and Sun 1997: 23).

**Genus *Boreophorbia* Michelsen, 1987**

*Boreophorbia hirtipes (Stein, 1907)

*Chirosia hirtipes* Stein.

Distribution in China. Qinghai (Stein 1907: 369; Hennig 1966: 63).

**Genus *Botanophila* Lioy, 1864**

Syn.: *Monochrotogaster* Ringdahl, 1932; *Pseudomyopina* Ringdahl, 1933.

Taxonomic note. Synonymy first proposed by Michelsen (2004) for *Monochrotogaster* and by Michelsen (1985: 39) for *Pseudomyopina*.

*Botanophila alcaecerca (Deng, 1997)

*Pegohylemyia alcaecerca* Deng.

Distribution. China: Shandong, Sichuan (Deng 1997: 201).

*Botanophila alishana Suwa, 1996

Distribution. China: Taiwan (Suwa 1996: 147).

*Botanophila angulisurstyla Xue & Zhang, 1996

Distribution. China: Qinghai (Xue and Zhang 1996b: 168).

*Botanophila angustisilva Xue & Yang, 2002

Distribution. China: Shaanxi, Gansu (Xue and Yang 2002: 73).

*Botanophila atricornis* (Fan & Wu, 1981)

*Monochrotogaster atricornis* Fan & Wu. Wei et al. 1998a: 702.

Distribution. China: Qinghai, Sichuan.

*Botanophila bicoloripennis Xue & Zhang, 1996

Distribution. China: Hebei, Sichuan (Xue and Zhang 1996b: 169).

*Botanophila caligotypa (Zheng & Fan, 1990)

*Pegohylemyia caligotypa* Zheng & Fan.

Distribution. China: Qinghai (Zheng and Fan 1990: 181).

*Botanophila cercodiscoides* (Fan, Zhong & Deng in Fan et al., 1988)

*Pegohylemyia okai cercodiscoides* Fan, Zhong & Deng.

*Botanophila okai cercodiscoides* (Fan, Zhong & Deng). Wei et al. 1998a: 693.

Distribution. China: Sichuan.

Taxonomic note. First ranked as species by Xue and Song (2007: 22).

*Botanophila chelonocerca Xue & Yang, 2002

Distribution. China: Gansu (Xue and Yang 2002: 74).

*Botanophila choui (Fan, Chen & Ma, 2000)

*Pegohylemyia choui* Fan, Chen & Ma.

Distribution. China: Qinghai (Fan et al. 2000: 130).

*Botanophila chui Suwa, 1996

Distribution. China: Taiwan (Suwa 1996: 140).

*Botanophila clavata (Hennig, 1970)

Distribution in China. Qinghai, Yunnan (Wang et al. 2006: 158).

*Botanophila convexifrons (Fan, Chen & Chen 1993)

*Pegohylemyia convexifrons* Fan, Chen & Chen.

Distribution. China: Henan, Xinjiang (Fan et al. 1993: 59).

*Botanophila cornuta (Deng, 1997)

*Pegohylemyia cornuta* Deng.

Distribution. China: Sichuan (Deng 1997: 202).

*Botanophila cuneata (Deng, Li & Liu, 1996)

*Pegohylemyia cuneata* Deng, Li & liu.

Distribution. China: Jiangxi, Sichuan (Deng et al. 1996: 427).

*Botanophila curvimargo (Zheng & Fan, 1990)

*Pegohylemyia curvimargo* Zheng & Fan.

Distribution. China: Qinghai (Zheng and Fan 1990: 181).

*Botanophila densispinula Xue & Song, 2007

Distribution. China: Sichuan (Xue and Song 2007: 25).

*Botanophila depressa* (Stein, 1907)

*Chortophila depressa* Stein.

*Botanophila oraria* (Collin, 1967). Wei et al. 1998a: 693.

Distribution in China. Qinghai, Gansu, Xizang (Stein 1907: 365; Wang et al. 2006: 158).

Taxonomic note. Synonymy first suggested by Hennig (1970: 392).

*Botanophila dianisenecio Xue & Wang, 2010

Distribution. China: Yunnan (Xue and Wang 2010: 457).

*Botanophila dolichocerca (Zheng & Fan, 1990)

*Pegohylemyia dolichocerca* Zheng & Fan.

Distribution. China: Heilongjiang, Qinghai (Zheng and Fan 1990: 182).

*Botanophila endotylata (Deng, Li & Liu, 1996)

*Pegohylemyia endotylata* Deng, Li & Liu.

Distribution. China: Henan, Sichuan (Deng et al. 1996: 426).

*Botanophila euryisurstyla (Deng, Liu & Li, 1995)

*Pegohylemyia euryisurstyla* Deng, Liu & Li.

Distribution. China: Sichuan (Deng et al. 1995: 375).

*Botanophila fanjingensis Wei, 2006

Distribution. China: Guizhou (Wei 2006b: 528).

*Botanophila flavibellula (Deng, Geng, Liu & Li, 1995)

*Pegohylemyia flavibellula* Deng Geng, Liu & Li.

Distribution. China: Jilin, Sichuan (Deng et al. 1995: 58).

*Botanophila fulgicauda (Deng, Liu & Li, 1995)

*Pegohylemyia fulgicauda* Deng, Liu & Li.

Distribution. China: Sichuan (Deng et al. 1995: 375).

*Botanophila fumidorsis* (Ackland, 1967)

*Pseudomyopina fumidorsis probola* Fan in Ye, Ni & Fan, 1982. Wei et al. 1998a: 702.

Distribution in China. Shandong, Gansu, Xinjiang.

Taxonomic note. Synonymy first proposed by Zhang and Zhu (2014: 23).

*Botanophila gnava (Meigen, 1826)

Distribution in China. Heilongjiang, Xinjiang (Wang et al. 2006: 159).

*Botanophila gnavoides (Hennig, 1970)

Distribution in China. Gansu, Xinjiang (Wang et al. 2006: 159).

*Botanophila guizhouensis Wei, 2006

Distribution. China: Guizhou (Wei 2006b: 529).

*Botanophila higuchii (Suwa, 1974)

*Pegohylemyia higuchii* Suwa.

Distribution in China. Shaanxi, Gansu (Wu and Zhang 1988: 348).

*Botanophila hohxiliensis Xue & Zhang, 1996

Distribution. China: Jilin, Qinghai (Xue and Zhang 1996b: 170).

*Botanophila kanmiyai Suwa, 1996

Distribution. China: Taiwan (Suwa 1996: 144).

*Botanophila latigena (Stein, 1907)

*Chortophila latigena* Stein.

*Pegohylemyia latigena* (Stein). Hennig 1970: 382.

*Botanophila latigena* (Stein). Wang et al. 2006: 159.

Distribution in China. Qinghai, Gansu, Hebei (Stein 1907: 359; Wang et al. 2006: 159).

*Botanophila latispinisternata Xue & Wang, 2010

Distribution. China: Yunnan (Xue and Wang 2010: 461).

*Botanophila ligoniformis (Deng, 1993)

*Pegohylemyia ligoniformis* Deng.

Distribution. China: Sichuan, Xizang (Deng 1993: 58).

*Botanophila longibarbata Xue & Wang, 2010

Distribution. China: Yunnan (Xue and Wang 2010: 455).

*Botanophila maculipes* (Zetterstedt, 1845)

*Botanophila pseudomaculipes* (Strobl, 1893). Wei et al. 1998a: 693.

Distribution in China. Sichuan, Xizang (Wang et al. 2006: 159).

Taxonomic note. Synonymy first proposed by Michelsen (1985: 51).

*Botanophila mediotubera (Deng, Li & Liu, 1996)

*Pegohylemyia mediotubera* Deng, Li & Liu.

Distribution. China: Jiangxi, Sichuan (Deng et al. 1996: 428).

*Botanophila melametopa* (Fan in Fan & Zheng, 1992)

*Pegohylemyia melametopa* Fan.

*Pegohylemyia nigrifrontata* Fan & Zheng, 1992.

*Botanophila melametopa* (Fan). Wei et al. 1998b: 2274.

*Botanophila nigrifrontata* (Fan & Zheng). Wei et al. 1998b: 2274.

Distribution. China: Sichuan.

Taxonomic note. Synonymy first proposed by Xue and Song (2007: 30).

*Botanophila menyuanensis (Zheng & Fan, 1990)

*Pegohylemyia menyuanensis* Zheng & Fan.

Distribution. China: Qinghai (Zheng and Fan 1990: 182).

*Botanophila monacensis (Hennig, 1970)

*Pegohylemyia monacensis* Hennig.

Distribution in China. Hebei (Zhao 1983: 24).

*Botanophila monoconica (Chen & Fan, 1995)

*Pegohylemyia monoconica* Chen & Fan.

Distribution. China: Qinghai (Chen and Fan 1995: 492).

*Botanophila nigribella (Deng, Geng, Liu & Li, 1995)

*Pegohylemyia nigribella* Deng, Geng, Liu & Li.

Distribution. China: Henan, Sichuan (Deng et al. 1995: 58).

*Botanophila pamirensis* (Ackland, 1967)

*Pseudomyopina pamirensis* Ackland. Wei et al. 1998a: 702.

Distribution in China. Henan, Xinjiang.

*Botanophila papiliocerca (Deng, 1997)

*Pegohylemyia papiliocerca* Deng.

Distribution. China: Shanxi, Sichuan (Deng 1997: 201).

Taxonomic note. *Pegohylemyia papiliocera* is an incorrect original spelling in the English summary (p. 204) by Deng (1997).

*Botanophila peltophora (Li, Cui & Fan, 1993)

*Pegohylemyia peltophora* Li, Cui & Fan.

Distribution. China: Neimenggu, Henan (Li et al. 1993: 129).

*Botanophila peninsularis Suh & Kwon, 1986

Distribution in China. Liaoning (Wang et al. 2006: 160).

*Botanophila pilicoronata Xue & Zhang, 1996

Distribution. China: Qinghai (Xue and Zhang 1996b: 171).

*Botanophila platysurstyla Xue & Song, 2007

Distribution. China: Sichuan (Xue and Song 2007: 26).

*Botanophila prenochirella (Zheng & Fan, 1990)

*Pegohylemyia prenochirella* Zheng & Fan.

Distribution. China: Qinghai (Zheng and Fan 1990: 182).

*Botanophila rotundivalva (Ringdahl, 1937)

Distribution in China. Shandong, Shaanxi (Xue and Yang 2002: 77; Wang et al. 2006: 160).

*Botanophila rubrigena (Schnabl, 1915)

Distribution in China. Qinghai, (Wang et al. 2006: 160).

*Botanophila rufifrons* (Fan & Chen, 1981)

*Monochrotogaster rufifrons* Fan & Chen. Wei et al. 1998a: 702.

Distribution. China: Qinghai.

*Botanophila sanctiforceps Xue & Yang, 2002

Distribution. China: Gansu (Xue and Yang 2002: 76).

*Botanophila sericea (Malloch, 1920)

*Botanophila sericea* (Malloch). Xue and Song (2007: 14).

Distribution in China. Not given.

*Botanophila spinisternatodea Xue & Wang, 2010

Distribution. China: Yunnan (Xue and Wang 2010: 460).

*Botanophila stenocerca (Zheng & Fan, 1990)

*Pegohylemyia stenocerca* Zheng & Fan.

Distribution. China: Qinghai (Zheng and Fan 1990: 182).

*Botanophila strictistriolata Xue & Zhang, 2005

Distribution. China: Gansu (Xue and Zhang 2005: 789).

*Botanophila submontivaga Xue & Zhang, 1996

Distribution. China: Gansu, Xinjiang (Xue and Zhang 1996a: 199).

*Botanophila subobscura Xue & Yang, 2002

Distribution. China: Gansu (Xue and Yang 2002: 77).

*Botanophila subspinulibasis Xue & Song, 2007

Distribution. China: Sichuan (Xue and Song 2007: 28).

*Botanophila tetracrula (Deng, 1997)

*Pegohylemyia tetracrula* Deng.

Distribution. China: Sichuan (Deng 1997: 203).

*Botanophila tortiforceps (Deng, 1993)

*Pegohylemyia tortiforceps* Deng.

Distribution. China: Sichuan (Deng 1993: 58).

*Botanophila trifurcata (Huckett, 1947)

Distribution in China. Qinghai, Sichuan (Wang et al. 2006: 161).

*Botanophila trifurcatoides Xue & Song, 1992

*Pegohylemyia trifurcata* Hennig, 1976 [preoccupied in *Botanophila*].

Distribution. China: Heilongjiang (Hennig 1976: 953).

*Botanophila trinivittata (Zheng & Fan, 1990)

*Pegohylemyia trinivittata* Zheng & Fan.

Distribution. China: Qinghai (Zheng and Fan 1990: 182).

*Botanophila unicolor* (Ringdahl, 1932)

*Monochrotogaster unicolor* Ringdahl. Wei et al. 1998a: 702.

Distribution in China. Xinjiang.

*Botanophila unicrucianella (Xue & Zhang, 1996)

*Pseudomyopina unicrucianella* Xue & Zhang.

Distribution. China: Qinghai (Xue and Zhang 1996b: 186).

*Botanophila unimacula Xue & Zhang, 1996

Distribution. China: Qinghai (Xue and Zhang 1996b: 172).

*Botanophila vicariola (Fan in Fan, Chen & Fang, 1987)

*Pegohylemyia vicariola* Fan.

Distribution. China: Xizang (Fan et al. 1987: 300).

*Botanophila zhuoniensis* (Jin, 1983)

*Pegohylemyia zhuoniensis* Jin.

*Botanophila zhuouniensis* [misspelling of *zhuoniensis*] (Jin). Wei et al. 1998a: 698.

Distribution. China: Gansu.

**Genus *Chirosia* Rondani, 1856**

Syn.: *Meliniella* Suwa, 1974; *Shakshainia* Suwa, 1974.

Taxonomic note. Synonymy first proposed by Michelsen (1988: 277).

*Chirosia bisinuata* (Tiensuu, 1939)

*Meliniella bisinuata* (Tiensuu). Wei et al. 1998a: 666.

Distribution in China. Heilongjiang.

*Chirosia forcipispatula Xue, 2001

Distribution. China: Yunnan (Xue 2001a: 307).

*Chirosia griseifrons* (Séguy, 1923)

*Meliniella griseifrons* (Séguy). Wei et al. 1998a: 666.

Distribution in China. Heilongjiang, Liaoning.

*Chirosia grossicauda* Strobl, 1899

*Chirosia parvicornis* (Zetterstedt, 1845) [misidentification]. Wei et al. 1998a: 656.

Distribution in China. Liaoning, Fujian.

Taxonomic note. The valid name for the present species first proposed by Michelsen (1985: 54).

*Chirosia nodula (Li, Cui & Fan, 1993)

*Meliniella nodula* Li, Cui & Fan.

Distribution. China: Henan (Li et al. 1993: 129).

*Chirosia rametoka* (Suwa, 1974)

*Shakshainia rametoka* Suwa. Wei et al. 1998a: 666.

Distribution in China. Heilongjiang, Liaoning.

*Chirosia spatuliforceps* (Fan & Chu in Fan, Chen, Ma & Ge, 1982)

*Meliniella spatuliforceps* Fan & Chu. Wei et al. 1998a: 666.

Distribution. China: Fujian, Sichuan, Yunnan.

*Chirosia strigilliformis* (Deng & Li, 1986)

*Meliniella strigilliformis* Deng & Li. Wei et al. 1998a: 666.

Distribution. China: Sichuan.

*Chirosia styloplasis (Zheng & Fan, 1990)

*Meliniella styloplasis* Zheng & Fan.

Distribution. China: Xizang (Zheng and Fan 1990: 181).

**Genus *Delia* Robineau-Desvoidy, 1830**

*Delia absidata Xue & Du, 2008

Distribution. China: Yunnan (Xue and Du 2008: 114).

*Delia ancylosurstyla Xue, 2002

Distribution. China: Gansu (Xue 2002: 73).

*Delia angustaeformis (Ringdahl, 1933)

Distribution in China. Xinjiang (Qian et al. 1998: 75).

*Delia apicifloralis Xue, 2002

Distribution. China: Gansu (Xue 2002: 74).

*Delia brevipalpis Xue & Zhang, 1996

Distribution. China: Qinghai (Xue and Zhang 1996b: 174).

*Delia conjugata Deng & Li, 1994

Distribution. China: Sichuan (Deng and Li 1994: 20).

*Delia conversatoides Xue & Zhang, 1996

Distribution. China: Qinghai (Xue and Zhang 1996b: 175).

*Delia diluta* (Stein, 1916)

*Delia diluta* (Stein). Fan et al. 1988: 178.

*Delia segmentata* (Wulp, 1896) [misidentification]. Wei et al. 1998a: 714.

Distribution in China. Qinghai.

Taxonomic note. Dely-Draskovits (1993: 49) listed by mistake *Delia diluta* as a junior synonym of the Nearctic *Delia segmentata*.

*Delia dovreensis Ringdahl, 1953

Distribution in China. Shanxi (Wang 1983: 412).

*Delia falciforceps Xue & Zhang, 1996

Distribution. China: Xinjiang (Xue and Zhang 1996a: 204).

*Delia fimbrifascia Xue & Du, 2009

Distribution. China: Yunnan (Xue and Du 2009: 155).

*Delia flavicommixta Xue & Zhang, 1996

Distribution. China: Xinjiang (Xue and Zhang 1996a: 206).

*Delia flavipes Tian & Ma, 1999

Distribution. China: Neimenggu (Tian and Ma 1999: 217).

*Delia flavogrisea (Ringdahl, 1926)

Distribution in China. Heilongjiang (Hennig 1974: 814).

*Delia floricola* Robineau-Desvoidy, 1830

*Delia floricola* Robineau-Desvoidy. Fan et al. 1988: 158.

*Delia cardui* (Meigen, 1826) [misidentification]. Wei et al. 1998a: 718.

Distribution in China. Xinjiang.

Taxonomic note. Dely-Draskovits (1993: 41) listed by mistake *Delia floricola* as a junior synonym of *Delia cardui*.

*Delia formosana Suwa, 1994

Distribution. China: Taiwan (Suwa 1994a: 63)

*Delia hohxiliensis Xue & Zhang, 1996

Distribution. China: Qinghai (Xue and Zhang 1996b: 176).

*Delia linearis* (Stein, 1898)

*Delia flabellifera* (Pandellé, 1900). Wei et al. 1998a: 718.

Distribution in China. Heilongjiang, Jilin, Neimenggu, Hebei, Shanxi, Gansu, Xinjiang.

Taxonomic note. Synonymy first proposed by Barták et al. (1990: 443) on advice from GCD Griffiths.

*Delia longiarista Xue, 2002

Distribution. China: Gansu (Xue 2002: 77).

*Delia longimastica Xue & Zhang, 1996

Distribution. China: Qinghai (Xue and Zhang 1996b: 177).

*Delia madoensis* Fan in Fan et al., 1988.

*Delia rondanii madoensis* Fan in Fan et al. Wei et al. 1998a: 723.

Distribution in China. Gansu, Qinghai.

Taxonomic note. First ranked as species by Zhang and Zhu (2014: 48).

*Delia mastigella Xue & Zhang, 1996

Distribution. China: Qinghai (Xue and Zhang 1996b: 178).

*Delia minutigrisea Xue & Zhang, 1996

Distribution. China: Qinghai (Xue and Zhang 1996b: 179).

*Delia nigriabdominis Xue, 2001

Distribution. China: Yunnan (Xue 2001a: 306).

*Delia parvicanalis Fan in Fan, Chen, Ma & Wu, 1984

Distribution. China: Qinghai, Sichuan (Fan et al. 1984: 243).

*Delia pectinator* Suwa, 1984

*Delia pectinator fuscilateralis* Fan in Fan & Zheng, 1992, syn. n. Wei et al. 1998b: 2269.

Distribution in China. Sichuan.

Taxonomic note. Griffiths (1993: 1444) showed that *Delia pectinator* described from Japan is widely distributed in northern North America. His redescription suggests that ssp. *fuscilateralis* Fan falls within the variation of *Delia pectinator*.

*Delia penicilliventris* Ackland, 2010

*Delia penicillaris* (Rondani, 1866) [misidentification]. Wei et al. 1998a: 722.

Distribution in China. Heilongjiang.

Taxonomic note. Ackland (2010: 80) showed that *Delia penicillaris* auct. consists of two different species of which true *Delia penicillaris* (Rondani) is only found in Central and South Europe.

*Delia persica Hennig, 1974

Distribution in China. Hebei (Zhao 1983: 24).

*Delia podagricicauda Xue, 1997

Distribution. China: Sichuan (Xue 1997: 1493).

*Delia scrofifacialis Xue & Zhang, 1996

Distribution. China: Qinghai (Xue and Zhang 1996b: 180).

*Delia stenostyla Deng & Li, 1994

Distribution. China: Sichuan (Deng and Li 1994: 20).

*Delia subatrifrons Xue & Du, 2009

Distribution. China: Sichuan, Yunnan (Xue and Du 2009: 157).

*Delia subinterflua Xue & Du, 2008

Distribution. China: Sichuan, Yunnan (Xue and Du 2008: 116).

*Delia taonura Deng & Li, 1994

Distribution. China: Sichuan (Deng and Li 1994: 18).

*Delia tenuiventris* (Zetterstedt, 1860)

*Delia conversata* (Tiensuu, 1936). Wei et al. 1998a: 714.

Distribution in China. Heilongjiang, Xinjiang.

Taxonomic note. Synonymy first proposed by Michelsen (1985: 58).

*Delia turcmenica Hennig, 1974

Distribution in China. Qinghai (Xue and Zhang 1996a: 201).

*Delia unguitigris Xue, 1997

Distribution. China: Sichuan (Xue 1997: 1494)

*Delia uralensis Hennig, 1974

Distribution in China. Heilongjiang, Jilin, Liaoning, Qinghai (Fan et al. 1988: 166).

**Genus *Egle* Robineau-Desvoidy, 1830**

*Egle ciliata* (Walker, 1849)

*Egle muscaria* (Fabricius, 1777) [misidentification]. Wei et al. 1998a: 660.

Distribution in China. Liaoning, Neimenggu.

Taxonomic note. The valid name for the present species was first proposed by Michelsen (1979: 193).

*Egle inermis* Ackland, 1970

*Egle steini* Schnabl, 1911 [misidentification]. Wei et al. 1998a: 661.

Distribution in China. Liaoning.

Taxonomic note. Misidentification first noted by Michelsen (2009: 42).

*Egle longirostris (Stein, 1907)

*Chortophila longirostris* Stein.

*Lasiomma longirostris* (Stein). Hennig 1972: 430.

*Egle longirostris* (Stein). Michelsen 1988: 277; Michelsen 2009: 20.

Distribution. China: Qinghai (Stein 1907: 366).

*Egle minuta* (Meigen, 1826)

*Egle korpokkur* Suwa, 1974. Wei et al. 1998a: 660.

*Egle gracilior* Zheng & Fan, 1990. Zheng and Fan 1990: 181.

Distribution in China. Liaoning, Shanxi, Gansu, Sichuan.

Taxonomic note. Synonymy first proposed by Michelsen (2004) for *Egle korpokkur* and by Griffiths (2003: 2350) for *Egle gracilior*.

*Egle subarctica* (Huckett, 1965)

*Egle cyrtacra* Fan & Wang in Fan, Chen, Ma & Ge, 1982. Wei et al. 1998a: 660.

Distribution in China. Shanxi.

Taxonomic note. Synonymy first proposed by Michelsen (2004).

**Genus *Emmesomyia* Malloch, 1917**

*Emmesomyia dorsalis (Stein, 1915)

*Chortophila dorsalis* Stein.

Distribution in China. Taiwan (Stein 1915: 47; Pont and Ackland 2009: 16, 55).

*Emmesomyia ovata (Stein, 1915)

*Chortophila ovata* Stein.

Distribution in China. Taiwan (Stein 1915: 47; Pont and Ackland 2009: 29, 56).

*Emmesomyia roborospinosa Cui, Li & Fan, 1993

Distribution. China: Heilongjiang (Cui et al. 1993: 137).

*Emmesomyia similata Suwa, 1991

Distribution in China. Guizhou (Wei 2006b: 533).

*Emmesomyia suwai* Ge & Fan, 1988

*Emmesomyia socia suwai* Ge & Fan. Wei et al. 1998a: 770.

Distribution in China. Heilongjiang, Henan, Sichuan, Guizhou, Yunnan.

Taxonomic note. First ranked as species by Suwa (1991: 20).

**Genus *Enneastigma* Stein, 1916**

Taxonomic note. The following two species are presently referred to *Enneastigma* rather than *Pegoplata*, because the male cerci that are not forming a projecting lobe between the surstyli, a derived character state only defining the genera *Pegoplata* and *Myopina* within the *Myopina* group of genera. In that respect *Enneastigma* agrees with *Calythea* Schnabl & Dziedzicki.

*Enneastigma fulva* (Malloch, 1934), COMB. NOV.

*Nupedia fulva* (Malloch). Wei et al. 1998a: 767.

Distribution in China. Zhejiang, Guangdong, Hainan, Sichuan, Guizhou, Yunnan.

*Enneastigma henanensis* (Ge & Fan, 1982), COMB. NOV.

*Nupedia henanensis* Ge & Fan. Wei et al. 1998a: 767.

Distribution. China: Henan, Sichuan, Guizhou, Yunnan.

*Enneastigma lengshanensis Xue, 2001, COMB. NOV.

*Pegoplata lengshanensis* Xue, 2001.

Distribution. China: Yunnan (Xue 2001b: 486).

**Genus *Eutrichota* Kowarz, 1893**

Syn.: *Eremomyia* Stein, 1898; *Pegomyza* Schnabl & Dziedzicki, 1911; *Arctopegomyia* Ringdahl, 1938; *Parapegomyia* Griffiths, 1984.

Taxonomic note. Synonymy first proposed by Griffiths (1984: 415) for *Eremomyia*, by Suwa (1974: 231) for *Pegomyza* and *Arctopegomyia*, and by Barták et al. (1990) for *Parapegomyia*.

*Eutrichota apiciserpenta Xue & Dong in Xue, Dong & Bai, 2012

Distribution. China: Yunnan (Xue et al. 2012: 81).

*Eutrichota breviungula Xue & Dong in Xue, Dong & Bai, 2012

Distribution. China: Sichuan, Yunnan, Xizang (Xue et al. 2012: 83).

*Eutrichota fanjingensis Wei, 2006

Distribution. China: Guizhou (Wei 2006b: 531).

*Eutrichota gansuensis (Xue & Zhang, 2005)

*Parapegomyia gansuensis* Xue & Zhang.

Distribution. China: Gansu (Xue and Zhang 2005: 796).

*Eutrichota latimana Xue & Zhang, 1996

Distribution. China: Qinghai (Xue and Zhang 1996b: 181).

*Eutrichota minutiungula Xue & Bai in Xue, Dong & Bai, 2012

Distribution. China: Sichuan (Xue et al. 2012: 84).

*Eutrichota nigriceps Xue & Zhang, 1996

Distribution. China: Qinghai (Xue & Zhang 1996b: 182).

*Eutrichota palaestinensis (Hennig, 1973)

Distribution in China. Shaanxi (Wu and Zhang 1988: 348).

*Eutrichota praepotens* (Wiedemann, 1817)

Distribution in China. Only record from northern China (Hennig 1972: 472) in need of verification.

*Eutrichota ruficeps Xue & Zhang, 1996

Distribution. China: Qinghai (Xue and Zhang 1996b: 183).

*Eutrichota turbida* (Huckett, 1951), SP. REV.

*Eremomyia triticiperda* (Stein, 1900) [misidentification]. Hennig 1972: 462 (in part: Kyrgyzstan).

*Eutrichota triticiperda* (Stein) [misidentification]. Griffiths 1984: 441.

*Eremomyia pilimana pilimarginata* Fan & Qian in Fan, Chen, Ma & Ge, 1982, syn. n.

*Eutrichota pilimarginata* (Fan & Qian). Wei et al. 1998a: 749.

Distribution in China. Xinjiang.

Taxonomic note. Comparative study has convinced one of us (VM) that *Eutrichota triticiperda* (Stein) from Central Europe is different from the species identified by that name from Central Asia (Hennig 1972: 462) and North America (Griffiths 1984: 446). The valid name for the Holarctic species is *Eutrichota turbida* (Huckett). It differs from *Eutrichota triticiperda* by the absence of a shiny area on the antenna and, in males only, by a different shape of sternite V and presence of a broad, shiny black median field on sternites II–IV.

**Genus *Hydrophoria* Robineau-Desvoidy, 1830**

Taxonomic note. *Hydrophoria* in the present narrow sense follows upon the recognition of *Zaphne* (q.v.) as a separate genus.

*Hydrophoria aberrans Stein, 1918

Distribution. China: Taiwan (Stein 1918: 159; Suwa 1985: 6)

*Hydrophoria fanjingensis Wei, 2006

Distribution. China: Guizhou (Wei 2006b: 525).

*Hydrophoria lushiensis Ge & Li, 1985

Distribution. China: Henan (Ge and Li 1985: 242).

*Hydrophoria nigrinitida Feng, 2006

Distribution. China: Sichuan (Feng 2006: 1).

*Hydrophoria robustisurstylus Feng, 2006

Distribution. China: Sichuan (Feng 2006: 1).

*Hydrophoria silvicola* (Robineau-Desvoidy, 1830)

*Hydrophoria annulata* (Pandellé, 1899) [misidentification]. Wei et al. 1998a: 672.

*Hydrophoria silvicola* (Robineau-Desvoidy). Xue et al. 2009: 421.

Distribution in China. Xinjiang, Liaoning, Jilin, Heilongjiang.

Taxonomic note. The valid name for the present species was first proposed by Barták et al. (1990: 443).

**Genus *Hylemya* Robineau-Desvoidy, 1830**

Syn.: *Lopesohylemya* Fan, Chen & Ma, 1989, syn. n.

Taxonomic note. Fan et al. (1989) established a new genus *Lopesohylemya* with the new species *Lopesohylemya qinghaiensis* (see below) as type species. They further suggested that their new genus should also accommodate the *histrio* species group of the genus *Eustalomyia* Kowarz. As noted in the discussion by Griffiths (1996: 1754), we disagree that *Lopeshylemyia* and *Eustalomyia* are closely related taxa. Instead, we propose that *Lopeshylemyia qinghaiensis* is closely related to *Hylemya flavicruralis* Suwa, 1989 described from Nepal. The distal articles of both antennae are missing in the holotype and only known specimen of *Lopesohylemya qinghaiensis*. Accordingly, the authors were unable to observe the plumose condition of the arista, a prime characteristic of the genus *Hylemya*.

*Hylemya teinosurstylia Xue & Zhang, 2004

Distribution. China: Guangxi, Yunnan (Xue and Zhang 2004: 546).

*Hylemya qinghaiensis* (Fan, Chen & Ma, 1989), COMB. NOV.

*Lopesohylemya qinghaiensis* Fan, Chen & Ma. Wei et al. 1998a: 732.

Distribution. China: Qinghai.

*Hylemya urbica* Wulp, 1896

*Hylemya latifrons* Schnabl. Wei et al. 1998a: 736.

Distribution in China. Heilongjiang.

Taxonomic note. Synonymy first published in Barták et al. (1990: 443) on advice from GCD Griffiths.

**Genus *Hylemyza* Schnabl & Dziedzicki, 1911**

Taxonomic note. First revived from synonymy with *Hylemya* Robineau-Desvoidy, 1830 by Michelsen (1985: 39).

*Hylemyza partita* (Meigen, 1826)

*Hylemya partita* (Meigen). Wei et al. 1998a: 736.

Distribution in China. Heilongjiang, Qinghai.

**Genus *Hyporites* Pokorny, 1893**

Syn.: *Engyneura* Stein, 1907

Taxonomic note. Species of *Engyneura* agree closely with those of *Hyporites*, and together they constitute a well defined, clearly monophyletic entity of anthomyiid flies. This was realized by Hennig (1966: 77), but he desisted from formally synonymizing these genera because no material of *Engyneura* was available to him. This synonymy was first proposed by Michelsen (2004).

*Hyporites curvostylata* (Fan & Chen in Fan, Chen, Fan, Ma & Zhong, 1980)

*Engyneura curvostylata* Fan & Chen. Wei et al. 1998a: 744.

Distribution. China: Qinghai.

*Hyporites gracilior* (Fan & Zhong in Fan, Chen, Fan, Ma & Zhong, 1980)

*Engyneura gracilior* Fan & Zhong. Wei et al. 1998a: 744.

Distribution. China: Qinghai, Xizang.

*Hyporites leptinostylata* (Fan, Van & Ma in Fan, Chen, Fan, Ma & Zhong, 1980)

*Engyneura leptinostylata* Fan, Van & Ma. Wei et al. 1998a: 744.

Distribution. China: Qinghai.

*Hyporites pilipes* (Stein, 1907)

*Engyneura pilipes* Stein. Wei et al. 1998a: 744.

Distribution. China: Gansu, Qinghai.

*Hyporites setigera* (Stein, 1907)

*Engyneura setigera* Stein. Wei et al. 1998a: 744.

Distribution in China. Gansu, Qinghai.

*Hyporites setifemorata* (Fan in Fan & Zheng, 1992)

*Engyneura setifemorata* Fan. Wei et al. 1998b: 2270.

Distribution. China: Sichuan.

*Hyporites yuanyea (Xue & Liu, 2013)

*Engyneura yuanyea* Xue & Liu.

Distribution. China: Yunnan (Xue and Liu 2013: 147).

**Genus *Lasiomma* Stein, 1916**

Syn.: *Acrostilpna* Ringdahl, 1929; *Sinohylemya* Hsue, 1980.

Taxonomic note. Synonymy first established by Griffiths (2003: 2380) for *Acrostilpna* and by Michelsen (1988: 276) for *Sinohylemya*.

*Lasiomma craspedodontum* (Hsue, 1980)

*Sinohylemya craspedodonta* Hsue. Wei et al. 1998a: 743.

Distribution in China. Jilin, Liaoning, Sichuan.

*Lasiomma latipenne* (Zetterstedt, 1838)

*Acrostilpna latipennis* (Zetterstedt). Wei et al. 1998a: 653.

Distribution in China. Heilongjiang.

*Lasiomma monticola* Suh & Kwon, 1985

*Sinohylemya ctenocnema* Hsue, 1980 [preoccupied in *Lasiomma*]. Wei et al. 1998a: 743.

Distribution in China. Heilongjiang, Liaoning.

Taxonomic note. Synonymy first proposed by Michelsen (2004), valid name by Suwa (2005: 100).

*Lasiomma picipes* (Meigen, 1826)

*Lasiomma octoguttatum* (Zetterstedt, 1845). Wei et al. 1998a: 662.

Distribution in China. Xizang.

Taxonomic note. Synonymy first proposed by Barták et al. (1990: 443).

*Lasiomma replicatum* (Huckett, 1929)

*Acrostilpna montana* Ma, 1988. Wei et al. 1998a: 653.

Distribution in China. Heilongjiang, Liaoning, Shanxi.

Taxonomic note. Synonymy first proposed by Griffiths (2003: 2406).

**Genus *Leucophora* Robineau-Desvoidy, 1830**

*Leucophora dasyprosterna* Fan & Qian in Fan et al. 1988

*Leucophora brevifrons dasyprosterna* Fan & Qian. Wei et al. 1998a: 728.

Distribution. China: Xinjiang.

Taxonomic note. First ranked as species by Zhang and Zhu (2014: 10).

*Leucophora liaoningensis Zhang & Zhang, 1998

Distribution. China: Liaoning (Zhang and Zhang 1998: 103).

*Leucophora obtusa (Zetterstedt, 1838)

Distribution in China. Liaoning (Fan and Zheng 1992: 1140).

*Leucophora xinjiangensis Xue & Zhang, 1996

Distribution. China: Xinjiang (Xue and Zhang 1996a: 209).

**Genus *Mycophaga* Rondani, 1856**

*Mycophaga testacea (Gimmerthal, 1834)

Distribution in China. Sichuan (Feng et al. 2010: 35).

**Genus *Myopina* Robineau-Desvoidy, 1830**

*Myopina myopina (Fallén, 1824)

Distribution in China. Shanxi (Wang 1995: 94).

**Genus *Paradelia* Ringdahl, 1933**

Syn.: *Pseudonupedia* Ringdahl, 1959.

Taxonomic note. Synonymy first proposed by Barták et al. (1990: 443). Incidentally, *Pseudonupedia* Ringdahl is unavailable, as no type species was designated. The name was first made available by Huckett (1971: 76) who proposed *Anthomyia intersecta* Meigen, 1826 as type species.

*Paradelia brunneonigra (Schnabl in Schnabl & Dziedzicki, 1911)

*Pseudonupedia brunneonigra* (Schnabl). Wu and Zhang 1988: 348.

Distribution in China. Shaanxi, Gansu.

*Paradelia intersecta* (Meigen, 1826)

*Pseudonupedia intersecta* (Meigen). Wei et al. 1998a: 761.

Distribution in China. Jilin, Shanxi, Gansu.

*Paradelia lundbeckii (Ringdahl, 1918)

Distribution in China. Sichuan (Deng and Li 1993: 9).

*Paradelia palpata* (Stein, 1906)

*Pseudonupedia trigonalis* (Karl). Wei et al. 1998a: 761.

Distribution in China. Qinghai.

Taxonomic note. Synonymy first proposed by Griffiths (1987: 766).

**Genus *Paregle* Schnabl, 1911**

Syn.: *Chionomyia* Ringdahl, 1933.

Taxonomic note. Synonymy first proposed by Suwa (1974: 92).

*Paregle vetula* (Zetterstedt, 1838)

*Chionomyia vetula* (Zetterstedt). Wei et al. 1998a: 703.

Distribution in China. Heilongjiang, Jilin, Liaoning, Neimenggu, Beijing, Hebei, Shanxi, Shandong, Henan.

**Genus *Pegomya* Robineau-Desvoidy, 1830**

*Pegomya acisophalla Xue, 2003

Distribution. China: Yunnan (Xue 2003: 80).

*Pegomya agilis Wei, 2006

Distribution. China: Guizhou (Wei 2006a: 286).

*Pegomya basichaeta Li, Liu & Fan in Li, Liu, Fan & Cui, 1999

Distribution. China: Henan (Li et al. 1999: 244).

*Pegomya calyptrata (Zetterstedt, 1846)

Distribution in China. Qinghai (Ci and Yang 1986: 26).

*Pegomya chaetostigmata Zheng & Fan, 1990

Distribution. China: Xizang (Zheng and Fan 1990: 182).

*Pegomya cricophalla Xue, 2003

Distribution. China: Yunnan (Xue 2003: 81).

*Pegomya deprimata (Zetterstedt, 1845)

Distribution in China. Jiangxi (Wu and Zhang 1988: 349).

*Pegomya diplothrixa Li, Liu & Fan in Li, Liu, Fan & Cui, 1999

Distribution. China: Henan (Li et al. 1999: 243).

*Pegomya flavifrons (Walker, 1849)

Distribution in China. Shanxi, Qinghai, Xinjiang (Song et al. 2007: 230)

*Pegomya heteroparamera Zheng & Fan, 1990

Distribution. China: Sichuan (Zheng and Fan 1990: 184).

*Pegomya hohxiliensis Xue & Zhang, 1996

Distribution. China: Qinghai (Xue and Zhang 1996b: 185).

*Pegomya huanglongensis Deng & Li, 1993

Distribution. China: Sichuan (Deng and Li 1993: 8).

*Pegomya incrassata Stein, 1907

*Pegomyia incrassata* Stein.

Distribution in China. Guangdong, Qinghai (Stein 1907: 356; Song et al. 2007: 230).

*Pegomya japonica* Suwa, 1974

*Pegomya japonica japonica* Suwa. Wei et al. 1998a: 789.

*Pegomya japonica mokanensis* Fan, 1982. Wei et al. 1998a: 789.

Distribution in China. Zhejiang, Fujian, Sichuan.

Taxonomic note. Synonymy first proposed by Zhang and Zhu (2014: 30).

*Pegomya lageniforceps Xue, 2003

Distribution. China: Yunnan (Xue 2003: 82).

*Pegomya lurida* (Zetterstedt, 1846)

*Pegomya valgenovensis* Hennig. Wei et al. 1998a: 798.

Distribution in China. Heilongjiang, Jilin, Liaoning, Sichuan.

Taxonomic note. Synonomy first proposed by Michelsen (2004).

*Pegomya mediarmata Zheng & Xue, 2002

Distribution. China: Liaoning (Zheng and Xue 2002: 159).

*Pegomya mirabifurca Cui, Li & Fan, 1993

Distribution. China: Heilongjiang, Henan, Neimenggu (Cui et al. 1993: 137; Song et al. 2007: 230).

*Pegomya nigripraepeda Feng, 2006

Distribution. China: Sichuan (Feng 2006: 2).

*Pegomya nudapicalis* Li & Deng in Fan et al., 1988

*Pegomya dichaetomyiola nudapicalis* Li & Deng. Wei et al. 1998a: 784.

Distribution. China: Sichuan.

Taxonomic note. *Pegomya dichaetomyiola nudicpiculis* and *Pegomya dichaetomyiola nudiapicalis* are incorrect original spellings in the Index (p. 391) and in the English summary (p. 396) by Fan et al. (1988). Ranked as species by Zhang and Zhu (2014: 19).

*Pegomya pulchripes (Loew, 1857)

Distribution in China. Hebei, Sichuan (Zhao 1983: 24).

*Pegomya revolutiloba Zheng & Fan, 1990

Distribution. China: Xizang (Zheng and Fan 1990: 184).

*Pegomya rhagolobos* Li, Deng, Zhu & Sun, 1984

*Pegomya rhagolobs* [misspelling of *rhagolobos*] Li, Deng, Zhu & Sun. Wei et al. 1998a: 788.

Distribution. China: Sichuan.

*Pegomya rufina (Fallén, 1825)

Distribution in China. Shaanxi (Wu and Zhang 1988: 349).

*Pegomya semicircula Li, Liu & Fan, 1999

Distribution. China: Henan (Li et al. 1999: 243).

*Pegomya setaria (Meigen, 1826)

Distribution in China. Shanghai (Hennig 1973: 635).

*Pegomya spiraculata Suwa, 1974

Distribution in China. Liaoning (Song et al. 2007: 230).

*Pegomya sublurida Hsue, 1981

Distribution. China: Liaoning (Hsue 1981: 89).

*Pegomya tabida (Meigen, 1826)

Distribution in China. Shaanxi (Wu and Zhang 1988: 349).

*Pegomya unilongiseta Fan & Huang in Fan, Huang, Zou & Wu, 1984

Distribution. China: Fujian (Fan et al. 1984: 220).

*Pegomya yunnanensis Xue, 2001

Distribution. China: Yunnan (Xue 2001b: 487).

**Genus *Pegoplata* Schnabl & Dziedzicki, 1911**

Syn.: *Nupedia* Karl, 1930.

Taxonomic note. Griffiths (1986: 610) first proposed a wider concept of *Pegoplata* to include species previously recognized in *Nupedia*.

*Pegoplata aestiva* (Meigen, 1826)

*Nupedia aestiva* (Meigen). Wei et al. 1998a: 765.

Distribution in China. Shanxi, Qinghai, Xinjiang, Sichuan, Yunnan, Xizang.

*Pegoplata annulata* (Pandellé, 1899)

*Pegoplata juvenilis* (Stein, 1898) [misidentification]. Wei et al. 1998a: 768.

Distribution in China. Heilongjiang.

Taxonomic note. Griffiths (1986: 622) recognized two subspecies under *Pegoplata juvenilis* (Stein, 1898), of which the nominal subspecies is Nearctic in distribution and its Palearctic counterpart was named as *Pegoplata juvenilis nitidicauda* (Schnabl, 1911). Barták et al. (1990: 448) and subsequent European authors treat the Palearctic taxon as a distinct species by the name *Pegoplata annulata* (Pandellé, 1899).

*Pegoplata infirma* (Meigen, 1826)

*Nupedia infirma* (Meigen). Wei et al. 1998a: 765.

Distribution in China. Heilongjiang, Hebei, Shanxi, Gansu, Xinjiang.

*Pegoplata laotudingga Zheng & Xue, 2002

Distribution. China: Liaoning (Zheng and Xue 2002: 158).

*Pegoplata linotaenia* (Ma, 1986)

*Nupedia linotaenia* Ma. Wei et al. 1998a: 767.

Distribution. China: Heilongjiang, Liaoning, Neimenggu.

*Pegoplata nigroscutellata* (Stein, 1920)

*Nupedia nigroscutellata* (Stein). Wei et al. 1998a: 767.

Distribution in China. Heilongjiang, Qinghai.

*Pegoplata patellans* (Pandellé, 1900)

*Nupedia patellans* (Pandellé). Wei et al. 1998a: 767.

Distribution in China. Gansu, Qinghai, Xinjiang, Sichuan.

*Pegoplata plicatura* (Hsue, 1981)

*Nupedia plicatura* Hsue. Wei et al. 1998a: 767.

Distribution. China: Liaoning.

*Pegoplata qiandianensis Wei, 2006

Distribution. China: Guizhou (Wei 2006b: 534).

**Genus *Phorbia* Robineau-Desvoidy, 1830**

*Phorbia fani Xue, 2001

Distribution. China: Sichuan, Yunnan (Xue 2001b: 485).

*Phorbia genitalis* (Schnabl, 1911)

*Phorbia securis xibeina* Wu, Zhang & Fan in Fan et al., 1988. Wei et al. 1998a: 742.

Distribution in China. Shaanxi, Gansu, Qinghai.

Taxonomic note. Synonymy first proposed by Ackland (1993: 213).

*Phorbia lobata* (Huckett, 1929)

*Phorbia perssoni* Hennig, 1976. Wei et al. 1998a: 742.

Distribution in China. Xinjiang.

Taxonomic note. Synonymy first proposed by Ackland (1993: 220) on advice from GCD Griffiths.

*Phorbia longipilis (Pandellé, 1900)

Distribution in China. Heilongjiang (Hennig 1976: 950).

*Phorbia morulella Fan, Li & Cui, 1993

Distribution. China: Henan (Fan et al. 1993: 133).

*Phorbia polystrepsis Fan, Chen & Ma, 2000

Distribution. China: Qinghai (Fan et al. 2000: 130).

*Phorbia simplisternita Fan, Li & Cui, 1993

Distribution. China: Henan (Fan et al. 1993: 133).

*Phorbia sinosingularis Zhang, Fan & Zhu, 2011

Distribution. China: Shanxi (Zhang et al. 2011: 298).

*Phorbia subcurvifolia Zhang, Fan & Zhu, 2011

Distribution. China: Heilongjiang (Zhang et al. 2011: 297).

*Phorbia subfascicularis* Suwa, 1994

*Phorbia fascicularis* Tiensuu, 1936 [misidentification]. Wei et al. 1998a: 740.

Distribution in China. Heilongjiang.

Taxonomic note. Misidentification first noted by Suwa (1994b: 535).

**Genus *Ringdahlia* Michelsen, 2014**

*Ringdahlia curtigena* (Ringdahl, 1935)

*Lasiomma curtigena* (Ringdahl). Wei et al. 1998a: 662.

Distribution in China. Gansu.

Taxonomic note. The genus *Ringdahlia* was established by Michelsen (2014: 12) for the present species previously referred to either *Lasiomma* Stein or *Chirosiomima* Hennig.

**Genus *Sinophorbia* Xue, 1997**

Syn.: *Sinophorbia* Xue in Wei et al., 1998

*Sinophorbia tergiprotuberans* Xue, 1997

*Sinophorbia tergiprotuberans* Xue in Wei et al., 1998b: 2301.

Distribution. China: Sichuan (Xue 1997: 1496).

Note. The present genus and species, intended for publication in “Flies of China” (Xue in Wei et al., 1998b), were accidentally published by Xue (1997: 1495–1497).

**Genus *Strobilomyia* Michelsen, 1988**

*Strobilomyia lijiangensis Roques & Sun in Roques, Sun, Zhang, Pan, Xu & Delplanque, 1996

Distribution. China: Yunnan (Roques et al. 1996: 421).

*Strobilomyia oriens (Suwa, 1983)

*Lasiomma abietes* [misspelling of *abietis*] (Huckett, 1953) [misidentification].

Distribution in China. Liaoning (Hsue 1983: 52).

Taxonomic note. Michelsen (1988: 312) noted that *Strobilomyia abietis* is a Nearctic species and replaced by *Strobilomyia oriens* in East Asia.

[*Strobilomyia sanyangi* Roques & Sun in Sun, Roques, Zhang & Xu, 1996]

Unavailable *nomen nudum* (Sun et al. 1996: 146).

*Strobilomyia sibirica Michelsen, 1988

Distribution in China. Heilongjiang (Roques et al. 2003: 365).

*Strobilomyia svenssoni Michelsen, 1988

Distribution in China. Heilongjiang (Sun et al. 1995: 10).

**Genus *Subhylemyia* Ringdahl, 1933**

Syn.: *Deliomyia* Fan in Fan et al., 1988, syn. n.

Taxonomic note. *Deliomyia* was proposed as a subgenus of *Subhylemyia* Ringdahl. The genus *Subhylemyia* is reasonably well defined and includes only two known species. Thus we see no reason to split this taxon any further.

*Subhylemyia dorsilinea* (Stein, 1920)

Subhylemyia (Deliomyia) lineola (Collin). Wei et al. 1998a: 734.

Distribution in China. Neimenggu, Qinghai.

Taxonomic note. Synonymy first proposed by Griffiths (1998: 1880).

**Genus *Zaphne* Robineau-Desvoidy, 1830**

Taxonomic note. First proposed by Michelsen (1985: 40) as valid generic name for a species group split off from *Hydrophoria* s. lat.

*Zaphne ambigua* (Fallén, 1823)

*Hydrophoria ambigua* (Fallén). Wei et al. 1998a: 672.

*Zaphne ambigua* (Fallén). Xue et al. 2009: 423.

Distribution in China. Heilongjiang.

*Zaphne divisa* (Meigen, 1826)

*Hydrophoria divisa* (Meigen). Wei et al. 1998a: 672.

*Zaphne divisa* (Meigen). Xue et al. 2009: 423.

Distribution in China. Heilongjiang, Neimenggu, Tianjin.

*Zaphne fasciculata* (Schnabl, 1915)

*Hydrophoria fasciculata* (Schnabl). Wei et al. 1998a: 672.

*Zaphne fasciculata* (Schnabl). Xue et al. 2009: 423.

Distribution in China. Heilongjiang.

*Zaphne ignobilis* (Zetterstedt, 1845)

*Hydrophoria ignobilis* (Zetterstedt). Wei et al. 1998a: 672.

*Zaphne ignobilis* (Zetterstedt). Xue et al. 2009: 424.

Distribution in China. Heilongjiang, Jinlin, Yunnan.

*Zaphne inuncta* (Zetterstedt, 1838)

*Hydrophoria hyalipennis* (Zetterstedt, 1855). Wei et al. 1998a: 672.

*Zaphne inuncta* (Zetterstedt). Xue et al. 2009: 424.

Distribution in China. Heilongjiang, Jilin.

Taxonomic note. Synonymy established by Michelsen (1985: 48, 49).

*Zaphne laxibarbiventris Xue & Dong in Xue, Bai & Dong, 2009

Distribution. China: Yunnan (Xue et al. 2009: 425).

*Zaphne lineatocollis* (Zetterstedt, 1838)

*Hydrophoria lineatocollis* (Zetterstedt). Wei et al. 1998a: 673.

*Zaphne lineatocollis* (Zetterstedt). Xue et al. 2009: 425.

Distribution in China. Heilongjiang.

*Zaphne maculipennis* (Stein, 1907)

*Hydrophoria maculipennis* Stein. Wei et al. 1998a: 673.

*Zaphne maculipennis* (Stein). Xue et al. 2009: 425.

Distribution in China. Neimenggu, Qinghai, Shanxi, Xizang.

*Zaphne melaena* (Stein, 1907)

*Hydrophoria melaena* Stein. Wei et al. 1998a: 673.

*Zaphne melaena* (Stein). Xue et al. 2009: 426.

Distribution in China. Heilongjiang, Liaoning, Qinghai, Neimenggu, Shanxi, Sichuan, Xinjiang.

*Zaphne nuda* (Schnabl in Schnabl & Dziedzicki, 1911)

*Hydrophoria nuda* (Schnabl). Wei et al. 1998a: 673.

*Zaphne nuda* (Schnabl). Xue et al. 2009: 427.

Distribution in China. Heilongjiang.

*Zaphne pullata (Wu, Liu & Wei, 1995)

*Hydrophoria pullata* Wu, Liu & Wei.

*Zaphne pullata* (Wu, Liu & Wei). Xue et al. 2009: 427.

*Hydrophoria disticrassa* Xue & Bai in Xue, Bai & Dong, 2009, syn. n.

Distribution. China: Guizhou, Yunnan (Wu et al. 1995: 290; Xue et al. 2009: 418).

Taxonomic note. Present synonymy based on comparison of the original illustrations of the male terminalia of the two nominal species.

*Zaphne tundrica* Schnabl in Schnabl & Dziedzicki, 1911

*Hydrophoria verticina* (Zetterstedt, 1838) [misidentification]. Wei et al. 1998a: 676.

*Zaphne verticina* (Zetterstedt) [misidentification]. Xue et al. 2009: 427.

Distribution in China. Xinjiang.

Taxonomic note. Misidentification first noted by Griffiths (1998: 1982).

*Zaphne venatifurca* (Zhong, 1985)

*Hydrophoria venatifurca* Zhong. Wei et al. 1998a: 676.

*Zaphne venatifurca* (Zhong). Xue et al. 2009: 427.

Distribution. Xizang.

*Zaphne ventribarbata* (Hsue, 1981)

*Hydrophoria ventribarbata* Hsue. Wei et al. 1998a: 676.

*Zaphne ventribarbata* (Hsue). Xue et al. 2009: 427.

Distribution. Jilin.

*Zaphne wierzejskii* (Mik, 1867)

*Hydrophoria wierzejskii* (Mik). Wei et al. 1998a: 676.

*Zaphne wierzejskii* (Mik). Xue et al. 2009: 427.

Distribution in China. Heilongjiang, Liaoning, Neimenggu, Shanxi, Qinghai, Xinjiang.

*Zaphne zetterstedtii* (Ringdahl, 1918)

*Hydrophoria zetterstedti* [misspelling of *zetterstedtii*] (Ringdahl). Wei et al. 1998a: 676.

*Zaphne zetterstedti* [misspelling of *zetterstedtii*] (Ringdahl). Xue et al. 2009: 428.

Distribution in China. Heilongjiang, Sichuan.

**Valid species removed from the list of Chinese Anthomyiidae**

*Eutrichota schineri* (Schnabl, 1910)

A record from NE China by Suwa (1999: 224) is mistaken and refers to *Eutrichota socculata* (Zetterstedt).

*Lasiomma seminitidum* (Zetterstedt, 1845)

Recorded from NE China by Suwa (1999: 224), but this refers to *Lasiomma craspedodontum* (Xue) as clarified by Suwa (2005: 93).

**Identity of *Heteroterma fanjingensis* Wei**

Wei (2006b: 531) proposed in the family Anthomyiidae a new genus *Heteroterma* for a new species *fanjingensis* based on 1 male, 1 female from Guizhou, China. On inspection of the original illustrations of the male and female terminalia it occurred to one of us (VM) that they might belong to a species of Scathophagidae rather than Anthomyiidae. Dr AL Ozerov, Zoological Museum, Moscow State University, was consulted and he immediately identified this nominal species that he did not know about beforehand. The nomenclatorial implications are summarized below.

**Genus *Scathophaga* Meigen, 1803**

Syn.: *Heteroterma* Wei, 2006, syn. n.

[name preoccupied by *Heteroterma* Gabb, 1869 in Tudiclidae, a fossil gastropod family]

*Scathophaga curtipilata* Feng, 2002

*Heteroterma fanjingensis* Wei, 2006, syn. n.

*Scatomyza fansipanicola* Ozerov in Ozerov & Krivosheina, 2011, syn. n.

Distribution. China: Sichuan, Guizhou; Vietnam (Feng 2002: 365; Wei 2006b: 531; Ozerov and Krivosheina 2011: 5).

Taxonomic note. Ozerov and Krivosheina (2011: 3) proposed a revival of the genus *Scatomyza* Fallén for a group of species previously recognized in *Scathophaga* Meigen. This is not followed here, as this may well result in a paraphyletic *Scatophaga* s. str.

## Discussion

We have attempted to consult all relevant publications on Chinese Anthomyiidae in the preparation of the above supplementary catalogue of Anthomyiidae covering both mainland China and Taiwan. The anthomyiid fauna of mainland China comprises 675 species in 37 genera which corresponds to more than one-third of the known world fauna and 84% of the currently recognized anthomyiid genera. One genus (*Sinophorbia* Xue, 1997) and c. 425 species of Anthomyiidae are presently regarded as endemic to mainland China; other 6 species are endemic to Taiwan. However, a revisional study of the difficult and species rich genus *Botanophila* in North America is still pending and may expectedly show that a substantial number of the species currently listed as endemic to China in reality are more widespread, northern Holarctic species.

**Figure 1.**
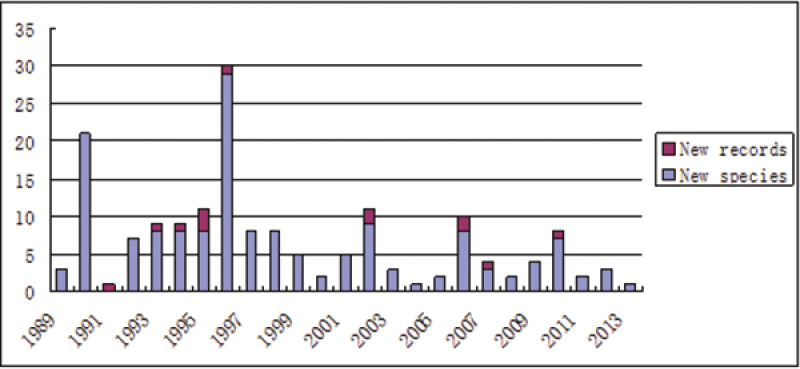
Number of anthomyiid species described or newly recorded from China and Taiwan all years from 1989 to 2013, a time span of 25 years.

As shown in the bar graph (Fig. 1), species have been described or newly recorded from mainland China all years since the publication of the regional monograph by Fan et al. (1988). The bar graph suggests that the knowledge about anthomyiid species diversity in China is still far from exhaustive. Predictably, many new species await discovery and description, especially from inaccessible high altitude regions such as the Tibetan Plateau.
